# On intra-supply chain system with an improved distribution plan, multiple sales locations and quality assurance

**DOI:** 10.1186/s40064-015-1498-1

**Published:** 2015-11-10

**Authors:** Singa Wang Chiu, Chao-Chih Huang, Kuo-Wei Chiang, Mei-Fang Wu

**Affiliations:** Department of Business Administration, Chaoyang University of Technology, Taichung, 413 Taiwan; Department of Industrial Engineering and Management, Chaoyang University of Technology, Taichung, 413 Taiwan; Department of Industrial Engineering and Systems Management, Feng Chia University, Taichung, 407 Taiwan

**Keywords:** Intra supply chain, Optimization, Multiple sales locations, Cost reduction distribution policy, Replenishment lot size

## Abstract

Transnational companies, operating in extremely competitive global markets, always seek to lower different operating costs, such as inventory holding costs in their intra- supply chain system. This paper incorporates a cost reducing product distribution policy into an intra-supply chain system with multiple sales locations and quality assurance studied by [Chiu et al., Expert Syst Appl, 40:2669–2676, (2013)]. Under the proposed cost reducing distribution policy, an added initial delivery of end items is distributed to multiple sales locations to meet their demand during the production unit’s uptime and rework time. After rework when the remaining production lot goes through quality assurance, *n* fixed quantity installments of finished items are then transported to sales locations at a fixed time interval. Mathematical modeling and optimization techniques are used to derive closed-form optimal operating policies for the proposed system. Furthermore, the study demonstrates significant savings in stock holding costs for both the production unit and sales locations. Alternative of outsourcing product delivery task to an external distributor is analyzed to assist managerial decision making in potential outsourcing issues in order to facilitate further reduction in operating costs.

## Background

In contemporary transnational companies, manufacturing task is often accomplished by a designated production unit, and then simultaneously transporting finished items to multiple sales offices in different regions around the world. Management of such an intra-supply chain system often would like to know the best production- shipment policy in order to minimize the total expected system costs. Schwarz (1973) studied a continuous review deterministic one- warehouse N-retailer inventory system with the objective of determining the optimal stocking policy to minimize the average system cost. Kim and Hwang (1988) examined a one supplier and multi-customer system with an incremental discount pricing schedule and single price break. For a single incremental discount system an algorithm is proposed for obtaining the optimal discount schedule. They also used numerical example to illustrate their algorithm for cases in which both the discount rate and the break point are unknown and either one is prescribed. Banerjee and Burton (1994) investigated the difference of coordinated and independent inventory replenishment policies for a vendor and multiple buyers system. A series of simulation experiments were conducted and the results indicated that classical lot sizing models do not adequately describe a situation where a single vendor produces and supplies a product to multiple industrial customers, buying in discrete lots. A common replenishment cycle-based, coordinated inventory control model is demonstrated to be superior to the independent optimization. Swenseth and Godfrey (2002) exhibited that the freight rate functions could be incorporated into inventory replenishment decisions without compromising the excellence of the decision nor will they increase unnecessary complexity to the decision process. Siajadi et al. (2006) considered a single-vendor multiple-buyer inventory model with a multiple- shipment policy. A methodology was proposed to derive the optimal shipment policy that minimizes the joint total relevant cost for both vendor and buyer(s) for such a joint economic lot size problem. Karabati and Sayin (2008) studied the coordination problem in a single-supplier multiple-buyer supply chain with quantity discounts. They modeled and discussed alternative efficiency of supplier–buyer costs sharing mechanisms, and proposed methods to design the associated discount schemes that take buyers’ expectations into account. Through numerical analysis of the coordination efficiency and allocation of the net savings of the proposed discount schemes, they showed that the supplier is able to coordinate the supply chain with high efficiency levels, and retain a significant portion of the net savings. Jha and Shanker (2013) examined a single-vendor multi-buyer integrated production-inventory model with controllable lead time and service level constraints. The demands from buyers are assumed to be independent normally distributed, and the lead time of every buyer can be reduced at an added crash cost. Inventory levels are reviewed by buyers using the continuous review policy, and the unsatisfied demand at the buyers is completely backordered. They developed a model considering service level constraint for each buyer and used a Lagrangian multiplier technique-based algorithm to derive the optimal production-inventory policy that minimizes the joint total expected cost of such a vendor-buyers system. Chiu et al. (2013) studied an intra-supply chain system in which a single production unit manufactures products to meet the demands of multiple regional sales offices and incorporates quality assurance into its production process. In their study, the considerations related to a product’s quality assurance include inspection of all units produced, rework of nonconforming items and failure in rework. A multi-shipment policy was used to synchronously deliver finished items to multiple locations in order to satisfy customer demands. An optimal production lot-size and shipment policy was determined for their proposed intra-supply chain system. Additional studies that addressed various aspects of quality assurance, multi- delivery, and multi-customers issues of vendor–buyer integrated systems can also be referred to (Khouja 2003; Benjaafar and Elhafsi 2006; Ervolina et al. 2009; Chiu and Chang 2014; Murugan and Selladurai 2014; Rodger 2014; Safaei 2014; Sana et al. 2014; Tseng et al. 2014; Wee et al. 2014).

This study incorporates a cost reduction *n* + 1 product distribution policy into an intra-supply chain system studied by Chiu et al. (2013) to facilitate lowering inventory holding costs for both the production unit and sales offices; thus, cutting down operating costs for such a specific intra-supply chain system. Detailed description and mathematical modeling of the proposed model are provided in next section.

## Description and mathematical modeling

To ease readability, in this study we use same notations as those used in mathematical modeling and formulation in Chiu et al.’s study (2013). Recall the problem description of their specific intra-supply chain system as follows. A product can be manufactured at an annual rate *P* by a single production unit, and an *x* portion of defective items may be randomly produced at a rate *d* during the production process. The unit manufacturing cost including the inspection cost is *C*. All defective items are reworked immediately after the regular production process ends in each cycle at a rate of *P*_1_, and there exists a rate of failure in rework *θ*_1_. To prevent shortages the production rate *P* must satisfy (*P* – *d* − *λ*) > *0*, where *λ* is the sum of the demands of all customers (i.e., the sum of *λ*_*i*_), and *d* can be expressed as *d* = *Px*. Cost parameters used in cost analysis include the following: unit holding cost *h*; set-up cost per production cycle *K*; unit cost *C*_R_ and unit holding cost *h*_1_ for each reworked item; unit disposal cost *C*_S_ for failures in rework; fixed delivery cost *K*_1*i*_ per shipment delivered to regional sales office *i*; unit holding cost *h*_2*i*_ for items retained by regional sales office *i*; and unit shipping cost *C*_Ti_ for items shipped to sales office *i*. Additional notations used in this study is listed in “Appendix A”.

Under the proposed delivery policy, an initial shipment of finished products is distributed to multiple sales locations to meet demand during the production unit’s uptime and rework time. After rework and once the remaining production lot goes through quality assurance, *n* fixed quantity installments of the finished products are transported to sales locations at a fixed time interval.

**Fig. 1:**
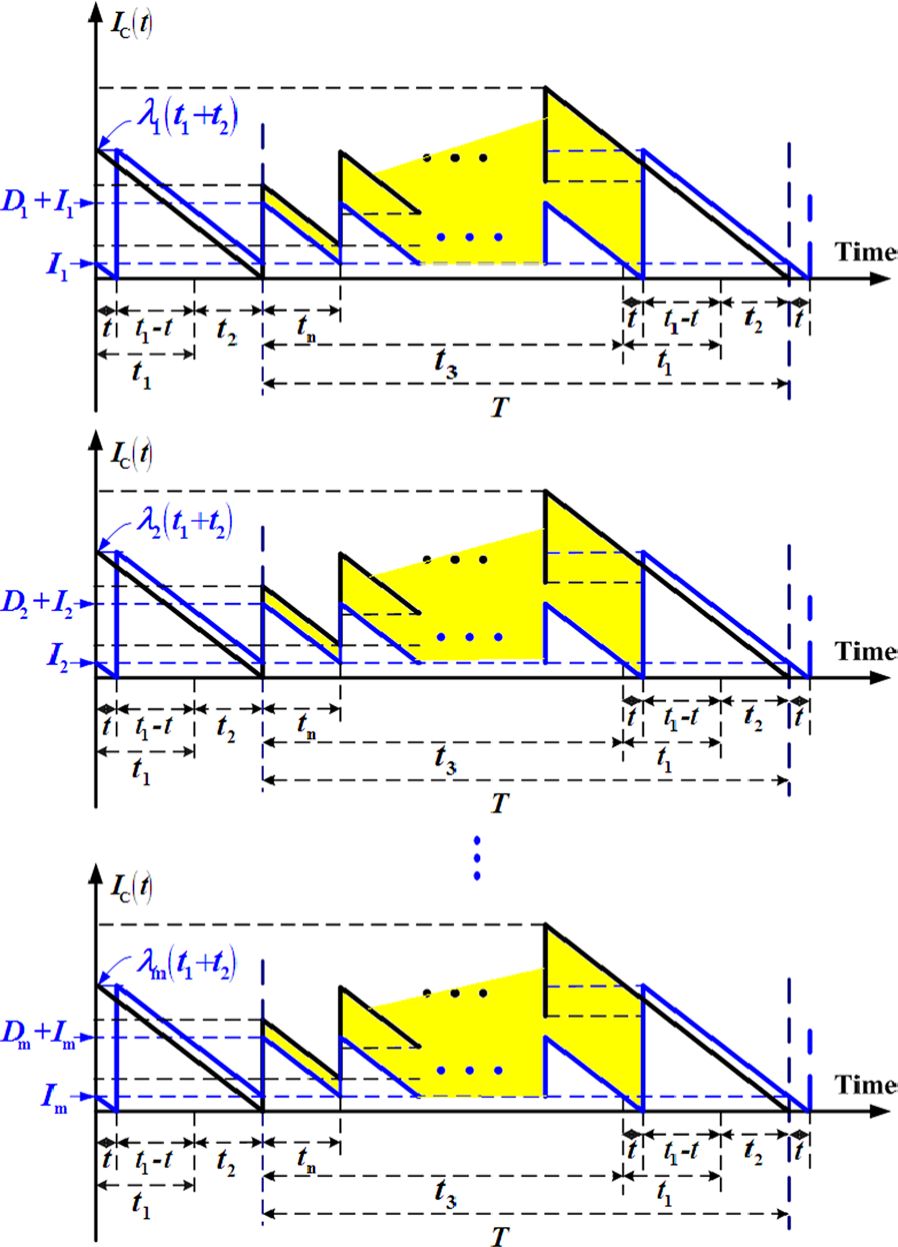
Expected reduction in sales offices’ stock holding costs (*yellow shaded area*) of the proposed model in comparison with that of Chiu et al. (2013)

Figure 1 depicts the expected reduction in sales officers’ stock holding costs (yellow shaded area) of the proposed model (in blue) in comparison with that of Chiu et al.’s model (2013) (in black).

Apply the similar mathematical techniques used in the conventional economic production quantity (EPQ) model (Hillier and Lieberman 2001; Nahmias 2009) and in the specific EPQ model with discontinuous delivery policy (Chiu et al. 2014) to the proposed model, total delivery costs can be obtained as

1 $$\left( {n + 1} \right)\sum\limits_{i = 1}^{m} {K_{1i} } + \sum\limits_{i = 1}^{m} {C_{{{\text{T}}i}} \left[ {Q\left( {1 - \theta_{1} x} \right)} \right]}$$

The variable holding costs at the producer’s side during the delivery time *t*_3_ where *n* fixed-quantity installments of the finished batch are delivered to retailers are

2 $$h\left( {\frac{n - 1}{2n}} \right)Ht_{3}$$

From Fig. 1, the total inventory holding costs for items stocked by retailers during the cycle are

3 $$\sum\limits_{i = 1}^{m} {h_{2i} } \left[ {\frac{{\lambda_{i} (t_{1} + t_{2} )^{2} }}{2} + n\left( {\frac{{D_{i} + 2I_{i} }}{2}} \right)t_{n} } \right]$$

Therefore, total production-inventory-delivery costs per cycle for the proposed *n* +1 delivery model, *TC*(*Q*, *n* + 1) consists of (1) the costs of variable manufacturing, setup, quality assurance, and inventory holding incurred in the production unit; (2) the costs for product distribution; and (3) the stock holding costs incurred in the sales offices, as follows:

4 $$\begin{gathered} TC\left( {Q,n{ + 1}} \right) = CQ + K + C_{R} \left[ {xQ} \right] + C_{S} \left[ {x\theta_{1} Q} \right] + \sum\limits_{i = 1}^{m} {C_{Ti} \left[ {Q\left( {1 - \theta_{1} x} \right)} \right]} + \left( {n + 1} \right)\sum\limits_{i = 1}^{m} {K_{1i} } \hfill \\ \, + h\left[ {\frac{{H_{1} }}{2}\left( t \right) + \frac{{H_{2} }}{2}\left( {t_{1} - t} \right) + \frac{{dt_{1} }}{2}\left( {t_{1} } \right) + \frac{{H_{2} + H}}{2}\left( {t_{2} } \right) + \left( {\frac{n - 1}{2n}} \right)Ht_{3} } \right] + h_{1} \cdot \frac{{dt_{1} }}{2} \cdot \left( {t_{2} } \right) \hfill \\ \, + \sum\limits_{i = 1}^{m} {h_{2i} } \left[ {\frac{{\lambda_{i} (t_{1} + t_{2} )^{2} }}{2} + n\left( {\frac{{D_{i} + 2I_{i} }}{2}} \right)t_{n} } \right] \hfill \\ \end{gathered}$$

By substituting all parameters in Eq. (4), applying the expected values of *x* to account for randomness of defective rate and with further derivations E[*TCU*(*Q*, *n* + 1)] is obtained as

5 $$\begin{gathered} E\left[ {TCU\left( {Q,n{ + 1}} \right)} \right] = C\lambda E_{3} + \frac{K\lambda }{Q}E_{3} + C_{R} \lambda E_{4} + C_{s} \theta_{1} \lambda E_{4} + \sum\limits_{i = 1}^{m} {C_{Ti} \lambda_{i} } + \frac{(n + 1)\lambda }{Q}E_{3} \sum\limits_{i = 1}^{m} {K_{1i} } \hfill \\ \, + \frac{hQ\lambda }{2}\left\{ \begin{aligned} \frac{{2\lambda^{2} }}{{P^{3} }}E_{0} + \frac{{4\lambda^{2} }}{{P^{2} P_{1} }}E_{1} + \frac{{2\lambda^{2} }}{{PP_{1}^{2} }}E_{2} - \frac{{\left( {1 - 2\theta_{1} E\left[ x \right]} \right)}}{P}E_{3} - \frac{\lambda }{{P^{2} }}E_{3} - \frac{2\lambda }{{PP_{1} }}E_{4} + \frac{1}{{E_{3} \lambda }} \hfill \\ - \frac{1}{{P_{1} }}\left[ {1 + \frac{\lambda }{{P_{1} }} - \theta_{1} } \right]E_{5} - \left( {\frac{1}{n}} \right)\left[ {\frac{1}{{E_{3} \lambda }} - \frac{2}{P} - \frac{2E\left[ x \right]}{{P_{1} }} + \frac{\lambda }{{P^{2} }}E_{3} + \frac{2\lambda }{{PP_{1} }}E_{4} + \frac{\lambda }{{(P_{1} )^{2} }}E_{5} } \right] \hfill \\ \end{aligned} \right\} \hfill \\ \, + \frac{{h_{1} Q\lambda }}{{2P_{1} }}E_{5} + \sum\limits_{i = 1}^{m} {h_{2i} } Q\lambda_{i} \left\{ \begin{aligned} \frac{\lambda }{{2P^{2} }}E_{3} + \frac{\lambda }{{PP_{1} }}E_{4} + \frac{\lambda }{{2P_{1}^{2} }}E_{5} + \frac{1}{{2n\lambda E_{3} }} - \frac{1}{nP} - \frac{E\left[ x \right]}{{nP_{1} }} + \frac{\lambda }{{P^{2} }}E_{6} + \frac{\lambda }{{PP_{1} }}E_{7} \hfill \\ + \frac{\lambda }{{2nP^{2} }}E_{3} + \frac{\lambda }{{nPP_{1} }}E_{4} - \frac{{\lambda^{2} }}{{P^{3} }}E_{0} + \frac{\lambda }{{2nP_{1}^{2} }}E_{5} - \frac{{\lambda^{2} }}{{PP_{1}^{2} }}E_{2} - \frac{{2\lambda^{2} }}{{P^{2} P_{1} }}E_{1} \hfill \\ \end{aligned} \right\} \hfill \\ \end{gathered}$$

where *E*_i_ denotes the following:

6 $$\begin{gathered} E_{0} = E\left( {\frac{1}{1 - x}} \right)E_{3} ; \, E_{1} = E\left( {\frac{x}{1 - x}} \right)E_{3} \, ; \, E_{2} = E\left( {\frac{{x^{2} }}{1 - x}} \right)E_{3} \, ; \, E_{3} = \frac{1}{{1 - \theta_{1} E\left[ x \right]}} \hfill \\ E_{4} = E\left[ x \right]E_{3} \, ; \, E_{5} = \left( {E\left[ x \right]} \right)^{2} E_{3} \, ; \, E_{6} = E\left( {\frac{1}{1 - x}} \right) \, ; \, E_{7} = E\left( {\frac{x}{1 - x}} \right) \hfill \\ \end{gathered}$$

### Proof of convexity and the optimal operating policy

To derive the optimal production-shipment policy for the proposed model, one must first prove that E[*TCU*(*Q*, *n* + 1)] is convex. The Hessian matrix equations (Rardin, 1998) are used to prove its convexity since the following equations are true (see “Appendix B” for details):

7 $$\left[ {\begin{array}{*{20}c} Q & n \\ \end{array} } \right] \cdot \left( {\begin{array}{*{20}c} {\frac{{\partial^{2} E\left[ {TCU\left( {Q,n{ + 1}} \right)} \right]}}{{\partial Q^{2} }}} & {\frac{{\partial^{2} E\left[ {TCU\left( {Q,n{ + 1}} \right)} \right]}}{\partial Q\partial n}} \\ {\frac{{\partial^{2} E\left[ {TCU\left( {Q,n{ + 1}} \right)} \right]}}{\partial Q\partial n}} & {\frac{{\partial^{2} E\left[ {TCU\left( {Q,n{ + 1}} \right)} \right]}}{{\partial n^{2} }}} \\ \end{array} } \right) \cdot \left[ {\begin{array}{*{20}c} Q \\ n \\ \end{array} } \right] = \frac{2\lambda }{Q}\frac{1}{{1 - \theta_{1} E\left[ x \right]}}\left( {K + \sum\limits_{i = 1}^{m} {K_{1i} } } \right) \, {\mathbf{ > }}{ 0}$$

The results of Eq. (7) are positive, because *λ*, *Q* (1 − *θ*_1_*E*[*x*]), *K,* and *K*_1*i*_ are all positive. Hence, E[*TCU*(*Q, n* + 1)] is a strictly convex function for all *Q* and *n* different from zero. So, the minimum of E[*TCU*(*Q, n* + 1)] exists. In order to derive the optimal lot size Q* and number of shipments n*, one can differentiate E[*TCU*(*Q, n* + 1)] with respect to *Q* and with respect to *n*, respectively, and solve the linear system of these equations [i.e., equations (B-1) and (B-3) in “Appendix B”] by setting these partial derivatives to zero. With further derivations, one obtains

8 $$Q^{*} = \sqrt { \, \frac{{2\left[ {K + \left( {n + 1} \right)\sum\limits_{i = 1}^{m} {K_{1i} } } \right]\lambda E_{3} }}{\begin{aligned} h\lambda \left\{ \begin{aligned} \frac{{2\lambda^{2} }}{{P^{3} }}E_{0} + \frac{{4\lambda^{2} }}{{P^{2} P_{1} }}E_{1} + \frac{{2\lambda^{2} }}{{P(P_{1} )^{2} }}E_{2} - \frac{{\left[ {1 - 2\theta_{1} E\left[ x \right]} \right]}}{P}E_{3} - \frac{\lambda }{{P^{2} }}E_{3} - \frac{2\lambda }{{PP_{1} }}E_{4} \hfill \\ - \frac{1}{{P_{1} }}\left[ {1 + \frac{\lambda }{{P_{1} }} - \theta_{1} } \right]E_{5} + \frac{1}{{E_{3} \lambda }} - \left( {\frac{1}{n}} \right)\left[ {\frac{1}{{E_{3} \lambda }} - \frac{2}{P} - \frac{2E\left[ x \right]}{{P_{1} }} + \frac{\lambda }{{P^{2} }}E_{3} + \frac{2\lambda }{{PP_{1} }}E_{4} + \frac{\lambda }{{P_{1}^{2} }}E_{5} } \right] \hfill \\ \end{aligned} \right\} \hfill \\ + \frac{{h_{1} \lambda }}{{P_{1} }}E_{5} + \sum\limits_{i = 1}^{m} {h_{2i} } \lambda_{i} \left\{ \begin{aligned} \frac{\lambda }{{P^{2} }}E_{3} + \frac{2\lambda }{{PP_{1} }}E_{4} + \frac{\lambda }{{P_{1}^{2} }}E_{5} + \frac{1}{{n\lambda E_{3} }} - \frac{2}{nP} - \frac{2E\left[ x \right]}{{nP_{1} }} + \frac{\lambda }{{P^{2} }}E_{6} + \frac{2\lambda }{{PP_{1} }}E_{7} \hfill \\ + \frac{\lambda }{{nP^{2} }}E_{3} + \frac{2\lambda }{{nPP_{1} }}E_{4} - \frac{{2\lambda^{2} }}{{P^{3} }}E_{0} + \frac{\lambda }{{nP_{1}^{2} }}E_{5} - \frac{{2\lambda^{2} }}{{PP_{1}^{2} }}E_{2} - \frac{{2\lambda^{2} }}{{P^{2} P_{1} }}E_{1} \hfill \\ \end{aligned} \right\} \hfill \\ \end{aligned} }}$$

and

9 $$n^{*} = \sqrt { \, \frac{{\left( {\sum\limits_{i = 1}^{m} {K_{1i} } + K} \right)\frac{1}{2}\left( {h\lambda - \sum\limits_{i = 1}^{m} {h_{2i} \lambda_{i} } } \right)\left[ {\frac{1}{{\lambda E_{3} }} - \frac{2}{P} - \frac{2E\left[ x \right]}{{P_{1} }} + \frac{\lambda }{{P^{2} }}E_{3} + \frac{2\lambda }{{PP_{1} }}E_{4} + \frac{\lambda }{{P_{1}^{2} }}E_{5} } \right]}}{{\left( { - \sum\limits_{i = 1}^{m} {K_{i} } } \right)\left\{ \begin{aligned} \frac{h\lambda }{2}\left\{ \begin{aligned} \frac{{2\lambda^{2} }}{{P^{3} }}E_{0} + \frac{{4\lambda^{2} }}{{P^{2} P_{1} }}E_{1} + \frac{{2\lambda^{2} }}{{PP_{1}^{2} }}E_{2} - \frac{{\left[ {1 - 2\theta_{1} E\left[ x \right]} \right]}}{P}E_{3} \hfill \\ - \frac{\lambda }{{P^{2} }}E_{3} - \frac{2\lambda }{{PP_{1} }}E_{4} - \frac{1}{{P_{1} }}\left[ {1 + \frac{\lambda }{{P_{1} }} - \theta_{1} } \right]E_{5} + \frac{1}{{E_{3} \lambda }} \hfill \\ \end{aligned} \right\} \hfill \\ + \frac{{h_{1} \lambda }}{{2P_{1} }}E_{5} + \sum\limits_{i = 1}^{m} {h_{2i} } \lambda_{i} \left\{ \begin{aligned} \frac{\lambda }{{2P^{2} }}E_{3} + \frac{\lambda }{{PP_{1} }}E_{4} + \frac{\lambda }{{2P_{1}^{2} }}E_{5} + \frac{\lambda }{{P^{2} }}E_{6} \hfill \\ + \frac{\lambda }{{PP_{1} }}E_{7} - \frac{{\lambda^{2} }}{{P^{3} }}E_{0} - \frac{{\lambda^{2} }}{{PP_{1}^{2} }}E_{2} - \frac{{2\lambda^{2} }}{{P^{2} P_{1} }}E_{1} \hfill \\ \end{aligned} \right\} \hfill \\ \end{aligned} \right\}}}}$$

The computational result of Eq. (9) does not necessarily have to be an integer number. However, the number of deliveries in real supply chain situations can only take on an integer value. In order to determine the integer value of n* that minimizes E[*TCU*(*Q, n* + 1)], two adjacent integers to *n* must be examined respectively. Let *n*^+^ denote the smallest integer greater than or equal to *n* [derived from Eq. (9)] and *n*^−^ denote the largest integer less than or equal to *n*. Substitute *n*^+^ and *n*^−^ respectively in Eq. (8) and then apply the results in Eq. (5), respectively. Choose the one that gives the minimum long-run average cost as the optimal replenishment- distribution policy.

## Numerical example

In order to simplify the comparison of the results, we use the same example as that used in Chiu et al.’s study (2013). Consider that a product can be manufactured at an annual rate *P* = 60,000 items by a single production unit in a transnational enterprise to meet the annual demands *λ*_*i*_ of its five regional sales offices, where *λ*_*i*_ is 200, 400, 600, 800, and 1000, respectively (i.e., the sum of all demands *λ* = 3000 items per year). During the production process, random nonconforming items are generated, and the defective rate seems to follow a uniform distribution over the interval [0, 0.3]. All nonconforming items are reworked at an annual rate of *P*_1_ = 3600 items at the end of regular production in each cycle. There exists a failure-in-rework rate *θ*_1_ = 0.2 during rework. A unit disposal cost *C*_S_ = $20 is associated with any item that fails rework. Other values of system parameters include *K* = $35,000; *K*_1*i*_ = $200, $250, $300, $350, and $400, for regional sales office *i* = 1, 2,…, and 5, respectively; *C* = $100; *h* = $25; *C*_R_ = $60; *h*_1_ = $60; *h*_2*i*_ = $85, $80, $75, $70 and $65, respectively; *C*_T*i*_ = $0.5, $0.4, $0.3, $0.2, and $0.1, respectively.

By applying Eq. (9), one obtains *n* = 5.272. By examining two adjacent integers to *n* and plugging them in Eq. (8), respectively, one obtains (*Q, n*^−^ + 1) = (2885,6) and (*Q, n*^+^ + 1) = (2980,7). Substitute these policies in Eq. (5), respectively, and choose the one that gives the minimum system cost, the optimal production-shipment policy (Q*, n* + 1) = (2885,6) is determined, and E[*TCU*(Q*, n* + 1)] = $434,009.

**Fig. 2:**
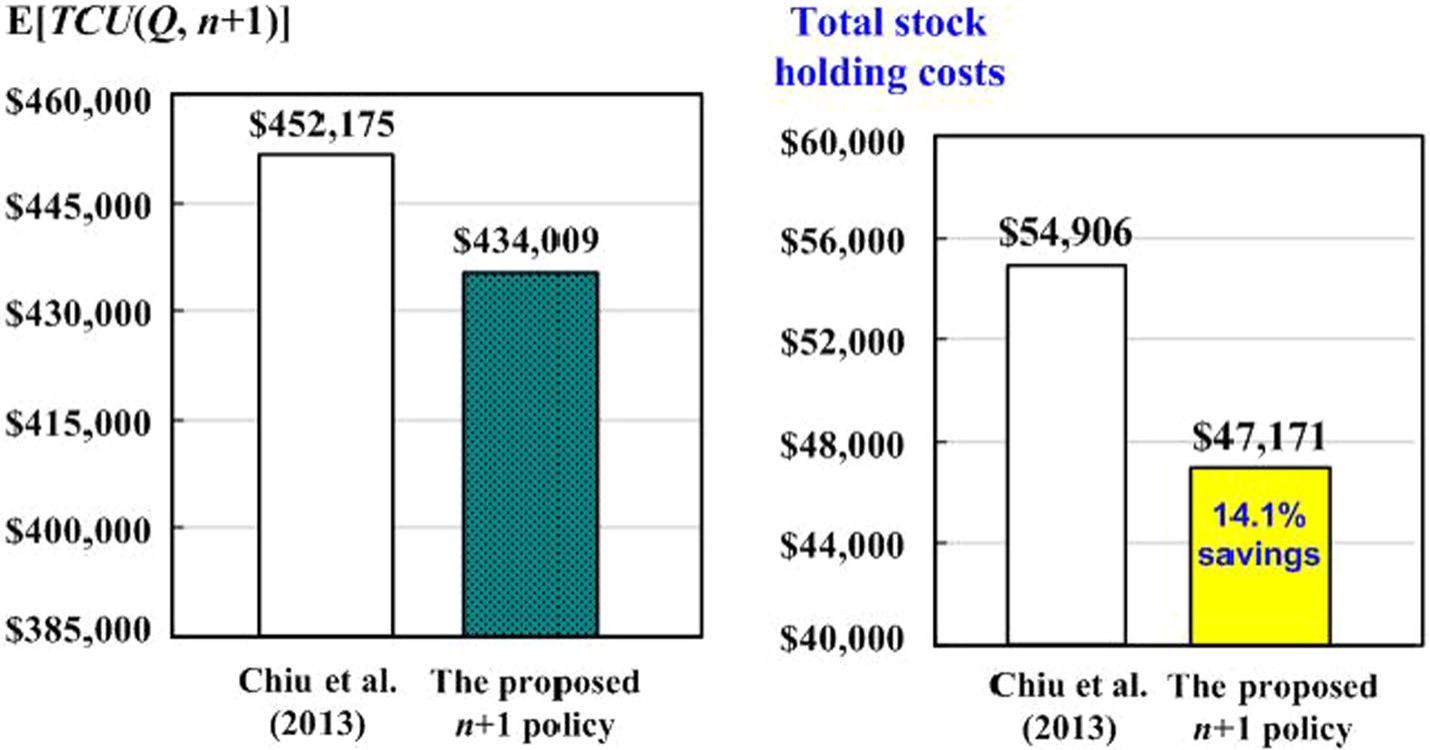
Comparisons of cost reductions on both the long-run average system cost and the total stock holding cost

Compared to E[*TCU*(Q* = 2337, n* = 5)] = $452,175 obtained in Chiu et al.’s model (2013), there is a $18,166, or 4.02 % saving in total system costs. The holding costs for both production unit and the sales offices combined are $47,171, as compared to $54,906 in Chiu et al.’s model (2013), resulting in a significant $7735 or 14.1 % savings. Figure 2 illustrates the savings on both the long-run average system costs and the total stock holding costs.

### Alternative of outsourcing product delivery task to an outside distributor

As mentioned in Chiu et al.’s study (2013), the tedious product delivery workload has low rewards for excellence, and the management of transnational firms may want to downsize the outbound logistics unit by outsourcing product delivery work to an external distributor. The proposed model can also provide a feasibility study on the outsourcing alternative. Recall total delivery cost for the proposed model is shown in Eq. (10):

10 $${\text{Total delivery cost in }}E\left[ {TCU\left( {Q,n + 1} \right)} \right] = \sum\limits_{i = 1}^{m} {C_{Ti} \lambda_{i} } + \frac{(n + 1)\lambda }{{Q\left( {1 - \theta_{1} E\left[ x \right]} \right)}}\sum\limits_{i = 1}^{m} {K_{1i} }$$

In this numerical example, by apply Eq. (10), we obtain total delivery cost $700 + $9,648 = $10,348. If the total contract costs for outsourcing delivery work are less than this amount, then evidently it is worthwhile hiring an outside distributor to take care of the product’s outbound logistics tasks. Consider the same contracted distributor’s cost schedule as used by Chiu et al. (2013), where fixed cost *K*_C_ = $9000 and variable cost *C*_TC_ = $0.25. With further analysis based on such a specific cost schedule, one finds the critical point *λ* = 5392 for determining whether or not to hire an external distributor.

**Fig. 3:**
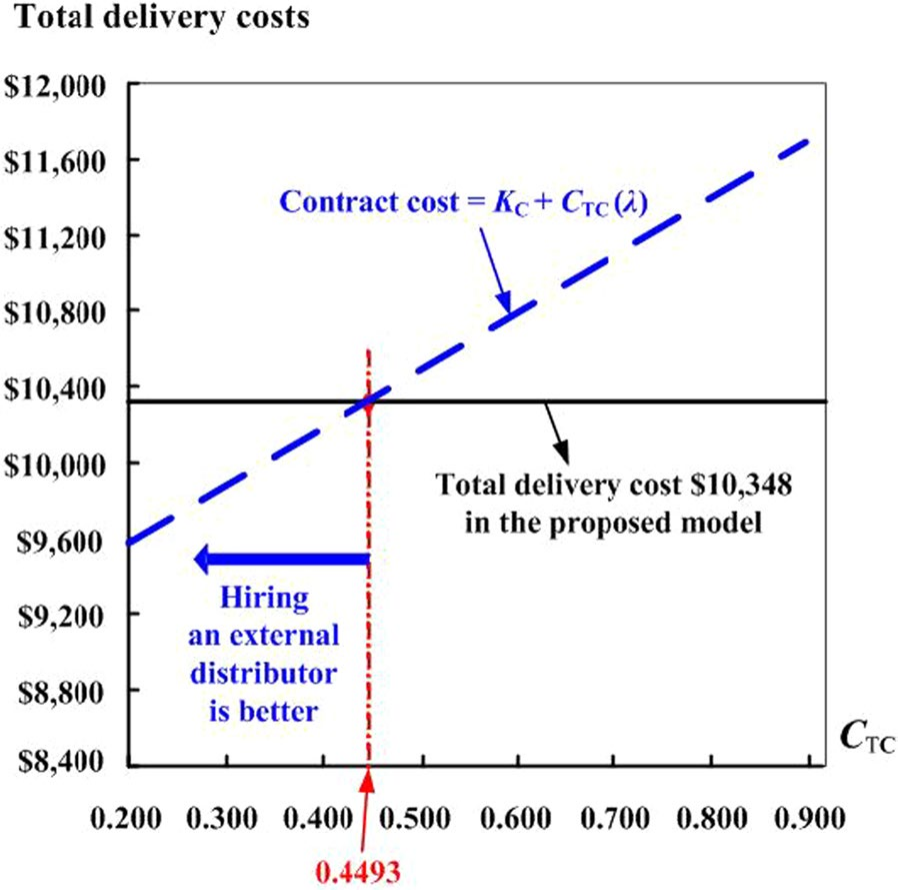
Variation of unit delivery cost *C*_TC_ effects on outsourcing decision making based on a specific contract cost schedule

Variation of unit delivery cost *C*_TC_ effects on outsourcing decision making is also depicted in Fig. 3. It indicates if unit delivery cost *C*_TC_ goes up to above $0.4493, the contract cost of outsourcing product delivery work to an external distributor can no longer be justified.

## Conclusions

Chiu et al. (2013) studied an intra-supply chain system with multiple sales locations and quality assurance, similar to the one that exists in present-day transnational enterprises. This study is an extension of their work as it incorporates a cost reducing (*n* + 1) delivery policy into such a specific intra-supply chain system with the purpose of lowering stock holding costs for both the production unit and sales offices. The optimal production lot size and number of deliveries that minimize total production-inventory-shipment cost for the proposed system are derived. Through a numerical example, we demonstrate significant savings in stock holding costs for both the production unit and sales locations. An analysis of outsourcing product delivery to an external distributor is also provided to assist the management of transnational firms in outsourcing decision making in order to further reduce their operating costs.

It is also noted that our research results are closed-form solutions [see Eqs. (8) and (9)] to the proposed problem, they are also valid for other numerical examples in wider environments. For future research, one interesting extension of the model will be to consider the effects of variable production rates on the optimal operating policies.

## Appendix A: Nomenclature

*P* = annual production rate for this specific product,

*Q* = production lot size per cycle—one of the decision variables,

*P*_1_ = annual reworking rate,

*n* = number of fixed quantity installments of the remaining lot to be delivered to sales offices after the rework (once the remaining lot is quality assured)—the other decision variable,

*θ*_1_ = portion of nonconforming items that fails during the reworking process,

*T* = production cycle length,

*t*_1_ = the production uptime,

*t*_2_ = time required for rework of nonconforming items in each cycle,

*t*_3_ = time required for delivering all remaining quality assured products in a lot to sales offices,

*H*_2_ = level of on-hand inventory in units for satisfying product demands during production unit’s production uptime *t*_1_ and rework time *t*_2_,

*H*_1_ = level of on-hand inventory in units when regular production process ends,

*H* = maximum level of on-hand inventory in units when the rework process ends,

*t*_n_ = a fixed interval of time between each installment of finished products delivered during *t*_3_,

*m* = number of regional sales offices,

*I*_c_(*t*) = the level of sales offices’ on-hand inventory at time *t*,

*D*_i_ = number of finished items being distributed to sales office *i* per delivery,

*I*_i_ = number of leftover items in sales office *i* after the depletion during t_*n*_,

*TC*(*Q*, *n* + 1) = total production-inventory-delivery costs per cycle for the proposed *n* +1 delivery model,

E[*TCU*(*Q*, *n* + 1)] = the expected total production-inventory-delivery costs per unit time for the proposed *n* + 1 delivery model.

## Appendix B

Details of computation of Eq. (7).

Applying the Hessian matrix equations, one obtains the following:

B-1 $$\begin{gathered} \frac{{\partial E\left[ {TCU\left( {Q,n + 1} \right)} \right]}}{\partial Q} = - \frac{1}{{Q^{2} }}\lambda \left[ {\left( {n + 1} \right)\sum\limits_{i = 1}^{m} {K_{1i} } + K} \right]E_{3} \hfill \\ \, \quad+ \frac{h\lambda }{2}\left\{ \begin{aligned} \frac{{2\lambda^{2} }}{{P^{3} }}E_{0} + \frac{{4\lambda^{2} }}{{P^{2} P_{1} }}E_{1} + \frac{{2\lambda^{2} }}{{PP_{1}^{2} }}E_{2} - \frac{{\left( {1 - 2\theta_{1} E\left[ x \right]} \right)}}{P}E_{3} - \frac{\lambda }{{P^{2} }}E_{3} - \frac{2\lambda }{{PP_{1} }}E_{4} + \frac{1}{{E_{3} \lambda }} \hfill \\ - \frac{1}{{P_{1} }}\left[ {1 + \frac{\lambda }{{P_{1} }} - \theta_{1} } \right]E_{5} - \left( {\frac{1}{n}} \right)\left[ {\frac{1}{{E_{3} \lambda }} - \frac{2}{P} - \frac{2E\left[ x \right]}{{P_{1} }} + \frac{\lambda }{{P^{2} }}E_{3} + \frac{2\lambda }{{PP_{1} }}E_{4} + \frac{\lambda }{{(P_{1} )^{2} }}E_{5} } \right] \hfill \\ \end{aligned} \right\} \hfill \\ \, \quad + \frac{{h_{1} \lambda }}{{2P_{1} }}E_{5} + \sum\limits_{i = 1}^{m} {h_{2i} } \lambda_{i} \left\{ \begin{aligned} \frac{\lambda }{{2P^{2} }}E_{3} + \frac{\lambda }{{PP_{1} }}E_{4} + \frac{\lambda }{{2P_{1}^{2} }}E_{5} + \frac{1}{{2n\lambda E_{3} }} - \frac{1}{nP} - \frac{E\left[ x \right]}{{nP_{1} }} + \frac{\lambda }{{P^{2} }}E_{6} + \frac{\lambda }{{PP_{1} }}E_{7} \hfill \\ + \frac{\lambda }{{2nP^{2} }}E_{3} + \frac{\lambda }{{nPP_{1} }}E_{4} - \frac{{\lambda^{2} }}{{P^{3} }}E_{0} + \frac{\lambda }{{2nP_{1}^{2} }}E_{5} - \frac{{\lambda^{2} }}{{PP_{1}^{2} }}E_{2} - \frac{{2\lambda^{2} }}{{P^{2} P_{1} }}E_{1} \hfill \\ \end{aligned} \right\} \hfill \\ \end{gathered}$$

B-2 $$\frac{{\partial E\left[ {TCU\left( {Q,n + 1} \right)} \right]}}{{\partial Q^{2} }} = \frac{2}{{Q^{3} }}\lambda \left[ {\left( {n + 1} \right)\sum\limits_{i = 1}^{m} {K_{1i} } + K} \right]E_{3}$$

B-3 $$\begin{aligned} \frac{{\partial E\left[ {TCU\left( {Q,n + 1} \right)} \right]}}{\partial n} &= \lambda \sum\limits_{i = 1}^{m} {K_{1i} } \frac{1}{Q}E_{3} + \left( {\frac{1}{{n^{2} }}} \right)\frac{1}{2}\left[ {hQ\lambda - \sum\limits_{i = 1}^{m} {h_{2i} } Q\lambda_{i} } \right] \hfill \\ &\quad\cdot \left[ {\frac{1}{{E_{3} \lambda }} - \frac{2}{P} - \frac{2E\left[ x \right]}{{P_{1} }} + \frac{\lambda }{{P^{2} }}E_{3} + \frac{2\lambda }{{PP_{1} }}E_{4} + \frac{\lambda }{{P_{1}^{2} }}E_{5} } \right] \hfill \\ \end{aligned}$$

B-4 $$\frac{{\partial E\left[ {TCU\left( {Q,n + 1} \right)} \right]}}{{\partial n^{2} }} = - \left( {\frac{1}{{n^{3} }}} \right)\left[ {hQ\lambda - \sum\limits_{i = 1}^{m} {h_{2i} } Q\lambda_{i} } \right]\left[ {\frac{1}{{E_{3} \lambda }} - \frac{2}{P} - \frac{2E\left[ x \right]}{{P_{1} }} + \frac{\lambda }{{P^{2} }}E_{3} + \frac{2\lambda }{{PP_{1} }}E_{4} + \frac{\lambda }{{P_{1}^{2} }}E_{5} } \right]$$

B-5 $$\begin{aligned} \frac{{\partial E\left[ {TCU\left( {Q,n + 1} \right)} \right]}}{\partial Q\partial n} &= - \lambda \sum\limits_{i = 1}^{m} {K_{1i} } \frac{1}{{Q^{2} }}E_{3} + \left( {\frac{1}{{n^{2} }}} \right)\frac{1}{2}\left[ {h\lambda - \sum\limits_{i = 1}^{m} {h_{2i} } \lambda_{i} } \right] \hfill \\ &\quad \cdot \left[ {\frac{1}{{E_{3} \lambda }} - \frac{2}{P} - \frac{2E\left[ x \right]}{{P_{1} }} + \frac{\lambda }{{P^{2} }}E_{3} + \frac{2\lambda }{{PP_{1} }}E_{4} + \frac{\lambda }{{P_{1}^{2} }}E_{5} } \right] \hfill \\ \end{aligned}$$

Substitute Eqs. (A-1) to (A-5) in the Hessian matrix equations (Rardin 1998) and with further derivations, one has Eq. (7) as follows:

B-6 $$\begin{aligned} \left[ {\begin{array}{*{20}c} Q & n \\ \end{array} } \right] \cdot &\left( {\begin{array}{*{20}c} {\frac{{\partial^{2} E\left[ {TCU\left( {Q,n{ + 1}} \right)} \right]}}{{\partial Q^{2} }}} & {\frac{{\partial^{2} E\left[ {TCU\left( {Q,n{ + 1}} \right)} \right]}}{\partial Q\partial n}} \\ {\frac{{\partial^{2} E\left[ {TCU\left( {Q,n{ + 1}} \right)} \right]}}{\partial Q\partial n}} & {\frac{{\partial^{2} E\left[ {TCU\left( {Q,n{ + 1}} \right)} \right]}}{{\partial n^{2} }}} \\ \end{array} } \right) \cdot \left[ {\begin{array}{*{20}c} Q \\ n \\ \end{array} } \right] \hfill \\ \, &= \frac{2\lambda }{Q}\frac{1}{{1 - \theta_{1} E\left[ x \right]}}\left( {K + \sum\limits_{i = 1}^{m} {K_{1i} } } \right) \, {\mathbf{ > }}{ 0} \hfill \\ \end{aligned}$$
